# Integrin α_D_β_2_ influences cerebral edema, leukocyte accumulation and neurologic outcomes in experimental severe malaria

**DOI:** 10.1371/journal.pone.0224610

**Published:** 2019-12-23

**Authors:** Isaclaudia G. de Azevedo-Quintanilha, Adriana Vieira-de-Abreu, André C. Ferreira, Patricia A. Reis, Tathiany I. Silva, Danielle de O. Nascimento, Robert A. Campbell, Vanessa Estato, Andrew S. Weyrich, Patrícia T. Bozza, Guy A. Zimmerman, Hugo C. Castro-Faria-Neto

**Affiliations:** 1 Laboratório de Imunofarmacologia, Instituto Oswaldo Cruz, Fundação Oswaldo Cruz, Rio de Janeiro, Rio de Janeiro, Brazil; 2 Department of Internal Medicine and Program in Molecular Medicine, University of Utah, Salt Lake City, Utah, United States of America; Ludwig-Maximilians-Universitat Munchen, GERMANY

## Abstract

Malaria is an infectious disease of major worldwide clinical importance that causes a variety of severe, or complicated, syndromes including cerebral malaria, which is often fatal. Leukocyte integrins are essential for host defense but also mediate physiologic responses of the innate and adaptive immune systems. We previously showed that targeted deletion of the α_D_ subunit (α_D_^-/-^) of the α_D_β_2_ integrin, which is expressed on key leukocyte subsets in mice and humans, leads to absent expression of the integrin heterodimer on murine macrophages and reduces mortality in mice infected with *Plasmodium berghei* ANKA (*P*. *berghei* ANKA). To further identify mechanisms involved in the protective effect of α_D_ deletion in this model of severe malaria we examined wild type C57BL/6 (WT) and α_D_^-/-^ mice after *P*. *berghei* ANKA infection and found that vessel plugging and leukocyte infiltration were significantly decreased in the brains of α_D_^-/-^ animals. Intravital microscopy demonstrated decreased rolling and adhesion of leukocytes in cerebral vessels of α_D_^-/-^ mice. Flow cytometry analysis showed decreased T-lymphocyte accumulation in the brains of infected α_D_^-/-^ animals. Evans blue dye exclusion assays demonstrated significantly less dye extravasation in the brains of α_D_^-/-^ mice, indicating preserved blood-brain barrier integrity. WT mice that were salvaged from *P*. *berghei* ANKA infection by treatment with chloroquine had impaired aversive memory, which was not observed in α_D_^-/-^ mice. We conclude that deletion of integrin α_D_β_2_ alters the natural course of experimental severe malaria, demonstrating previously unrecognized activities of a key leukocyte integrin in immune-inflammatory responses that mediate cerebral involvement.

## Introduction

Malaria remains the world’s most important parasitic disease and causes a spectrum of clinical involvement [1, 2]. In a minority of patients severe, or complicated malaria a constellation of syndromes with systemic manifestations and injury to critical organs are developed [2]. The most feared of these syndromes is cerebral malaria (CM), a serious and often fatal encephalopathy that is usually caused by in humans by *Plasmodium falciparum*. CM is most common in children in endemic regions, although nonimmune or semi-immune adults can also develop cerebral involvement [2, 3]. CM is lethal in 15–20% of individuals affected, and patients who survive may have debilitating long-term neurocognitive dysfunction [3–5]. Nevertheless, the biologic features that underly CM and other manifestations of severe malaria have not been adequately investigated or characterized.

Obstruction of cerebral vessels resulting from adhesion of infected red blood cells (iRBC) to the vascular endothelium is thought to be a central mechanism in CM [2, 3]. Sequestration of iRBC and their cytoadherence to the vascular endothelium are mediated by a variety of receptors including CD36, thrombospondin, VCAM-1, E-selectin, and endothelial protein C receptor [6–12]. Leukocytes and platelets sequestered in the microvasculature are also part of this vaso obstructive process [13–18]. Thus, CM is an inflammatory vasculopathy. In addition to vascular obstruction, CM is accompanied by an excessive production and release of inflammatory mediators. Among these mediators, TNF, IL-1β, MCP-1, and IFN-γ have been demonstrated to influence endothelial cell receptors, increasing iRBC cytoadherence and sequestration and promoting localization of activated leucocytes in the brains of infected animals in experimental models of severe malaria [19–23].

The leukocyte integrins are essential for host defense and mediate innate and adaptive immune responses via cell-cell and cell-extracellular matrix interactions that depend on specific ligand recognition. Binding of target ligands by leukocyte integrins not only tethers leukocytes to other cells and matrix but also delivers outside-in signals to intracellular pathways [24]. Adhesive interactions may play key roles in the pathophysiology of cerebral malaria, and leukocyte integrin activity is reported to be altered by malaria pigment [25–29]. Neverthelss, little is known about integrin expression and function in clinical or experimental malaria and the contributions of leukocytic integrins to severe complications of malarial infection.

The leukocyte integrins are a subfamily composed of four distinct members that are formed by α-subunits (α_L_ (CD11a), α_M_ (CD11b), α_X_ (CD11c), and α_D_ (CD11d)) in non-covalent association with a common β_2_-subunit (CD18) [24, 30–33]. Thus, these heterodimers are also called the CD18 or β_2_ integrins. Integrin α_D_β_2_ is the most-recently identified leukocyte integrin [33, 34]. It is highly expressed on human myeloid leukocyte subsets and murine macrophages and its pattern of distribution suggests contributions to atherosclerosis, rheumatoid arthritis, lung injury, and other pathologic conditions [34–39]. Furthermore, treatment with antibodies against the α_D_ subunit reduced intraspinal inflammation, oxidative damage, and free radical formation in rodent models of spinal cord injury [40–42].

We previously demonstrated that targeted deletion of α_D_ results in non-expression of the integrinα_D_β_2_, reduces mortality in an experimental model of severe malaria infection by *Plasmodium berghei* ANKA (*P*. *berghei* ANKA) that includes profound cerebral involvement, [37]. Part of the survival advantage appears to be due to reduction in acute lung injury, which also occurs in *P*. *berghei* ANKA infection [38, 43]. We now report that integrin α_D_β_2_ mediates events in the pathogenesis of cerebral involvement in this surrogate model of severe malaria, and that genetic deletion of α_D_ dramatically ameliorates neurological manifestations and outcomes.

## Materials and methods

### Mice and parasites

C57BL/6 wild type (α_D_^+/+^) and α_D_β_2_- deficient (α_D_^-/-^) mice [37] weighing 20-25g, littermate, were obtained from the Oswaldo Cruz Foundation breeding unit and used throughout the study. The animals were kept at constant temperature (25°C) with free access to food and water in a room with a 12-h light/dark cycle.

*Plasmodium berghei* ANKA (*P*. *berghei* ANKA) was maintained and provided by Dr. Leonardo de Moura Carvalho from Laboratório de Malária, Oswaldo Cruz Institute, Fiocruz, Rio de Janeiro, RJ, Brazil), and used as the infective parasite. The blood stage forms of the parasites were stored in liquid nitrogen after *in vivo* passages in C57BL/6 mice according to the protocol described elsewhere [27]. Mice were infected intraperitoneally (i.p.) with 10^5^ parasitized red blood cells and parasitemia was determined by direct light microscopy at different time points.

For cognitive impairment studies, the animals were inoculated intraperitoneally (i.p.) with 10^6^ parasitized red blood cells, inoculum that was provided in order to standardize clinical and behavioural signs of CM at day 6 post-infection, allowing intervention with antimalarial drugs.

### Ethics statement

The Animal Welfare Committee of the Oswaldo Cruz Institute approved the experiments in these studies under license number P-0528-08. The procedures described in this study were in accordance with the local guidelines and guidelines published in the National Institutes of Health Guide for the Care and Use of Laboratory Animals. The study is reported in accordance with the ARRIVE guidelines for reporting experiments involving animals.

### Brain edema

The permeability of the blood-brain barrier was determined at 7 days post infection (dpi) by intravenous injection of Evans blue dye 2% (w/v) solution in phosphate-buffered saline (PBS). One hour later the animals were sacrificed with terminal anesthesia by isoflurane and the vasculature was intracardiacaly perfused with 20 mL phosphate-buffered saline (PBS) using a peristaltic pump system. Brain tissue was placed in 3 mL of formamide at 56°C overnight to extract the Evans blue dye. The supernatant was read in a spectrophotometer at 620 nm [44].

### Isolation of brain leukocytes

Leukocytes were isolated from mouse brains as described [45]. Briefly, at 7 dpi, *P*. *berghei* ANKA-infected mice were sacrificed and by intracardiacaly perfused with phosphate-buffered saline (PBS) for 5 min using a peristaltic pump system to remove both circulating and non-adherent RBC and leukocytes. Brains were collected and placed in a 50 mL tube containing ice-cold PBS, 0,05% collagenase D (Sigma-Aldrich) and 2U/mL DNase I (Sigma-Aldrich). The brain was cut in small pieces and passed through a 70 mm cell strainer placed in a 10 cm Petri dish containing 10 mL of iced-cold PBS. The material was centrifuge for 10 mim at 390 g, resuspended in 20 mL of 1x PBS plus 30% Percoll, overlaid onto 70% Percoll and centrifuged at 390g for 20 min at room temperature. The cells were collected from the interface and washed twice with PBS before counting in Neubauer chambers and labeled for flow cytometry.

### Immunolabeling and flow cytometry

Cells were stained with appropriate dilutions of the following fluorochrome-labeled monoclonal antibodies (mAbs): PE-Cy5 anti-mouse CD8a (Ly-2 –clone 5–6.7) and FITC anti CD4 (L3T4 clone RM4-5) and then washed with PBS, fixed and analyzed by flow cytometry in a FACScalibur device (Becton Dickinson, USA). All reagents were purchased from Pharmingen/Becton-Dickinson (USA). Analyses were performed using CellQuest software. Cells were identified by their size (forward light scatter) and granularity (side light scatter) as previously described [46].

### Brain extracts and cytokine measurements

Excised brain tissues (0.1 g) were placed in 1 mL of homogenization buffer and macerated. The homogenates were subsequently frozen in liquid nitrogen. Prior to cytokine assay, samples were thawed and centrifuged at 6000 g for 20 min at 4°C and the supernatants were collected and used for cytokine measurements. Cytokines were analyzed using Luminex technology on the BioPlex system (Bio-Rad) using a mouse multiplex cytokine kit and assay protocols according to the manufacturer’s instructions (Upstate Biotechnology). Data analyses were performed with the Bio-Plex Manager software.

### Quantitative RT-PCR

Extraction of total RNA from brain and spleen was performed using TRIzol^®^ (Invitrogen-Carlsbad, CA, USA), according to the manufacturer’s instructions. After extraction, RNA concentration and quality were determined using a NanoDrop 2000 spectrophotometer (Thermo Scientific- Waltham, MA, USA). One microgram of total RNA was reverse-transcribed to single-strand cDNA using the SuperScript First-Stand (Invitrogen-Carlsbad, CA, USA). α_D_ transcripts in the cDNA pool obtained from the reverse transcriptase reaction were quantified by real-time quantitative fluorogenic PCR. TaqMan Universal PCR Master Mix (Applied Biosystems-Foster City, CA, USA) was used to quantify gene expression according to the manufacturer’s instructions. RNA expression levels were calculated using the Data Assist Software v.3, and normalized against the expression levels of the housekeeping gene hypoxanthine guanine phosphoribosyl transferase (HPRT) [37]. The primers used were as follows: α_D_ (TaqMan-murine-Mm01159115_m1) and HPRT (TaqMan-murine- Mm01545399_m1).

### Intravital microscopy

Intravital microscopy was performed in infected and non-infected mice of both genotypes. Animals were anaesthetised with an intraperitoneal mixture of ketamine (150 mg/kg; Laboratório Cristália, Itapira, SP, Brazil) and xylazine (10 mg/kg; Rompun^®^; Bayer, São Paulo, SP, Brazil). The tail vein was cannulated for the intravenous administration of fluorescent tracers and additional anesthesia. The core temperature was monitored with a rectal probe and maintained at 37 °C with a homoeothermic blanket system (Harvard Apparatus, Cambridge, UK). Intravital microscopy was performed as previously described [47]. Briefly, animals were fixed in a stereotaxic frame, the left parietal bone was exposed by a midline skin incision; a cranial window overlying the right parietal bone was created with a high-speed drill (Beltec, and the dura mater and the arachnoid membranes were excised and withdrawn to expose the cerebral microcirculation. The cranial window was suffused with artificial cerebrospinal fluid (in mmol: NaCl, 132; KCl, 2.95; CaCl2, 1.71; MgCl2, 0.64; NaHCO3, 24.6; dextrose, 3.71; and urea, 6.7; at 37 °C, pH 7.4). During this procedure, the core temperature was maintained at 37 °C with the homoeothermic blanket system (Harvard Apparatus, Cambridge, UK). Rhodamine 6G (0.3 mg/kg; Sigma, St. Louis, MO, USA) was injected via the tail vein cannula. The animals were then placed under an upright fixed-stage intravital microscope equipped with a mercury lamp (Olympus BX51/WI, NY, USA) coupled to a CCD digital video camera system (Optronics, TKY, Japan). Olympus 20X objectives were used in the experiments and produced total magnifications of 200X.

In order to evaluate leukocyte–endothelial interactions in post-capillary venules, rhodamine 6G-labeled leukocytes were visualized with a fluorescent light source (epi-illumination at 510–560 nm using a 590 nm emission filter). Rolling leukocytes were defined as white cells moving at a velocity less than that of erythrocytes and expressed as number of cells/min). Leukocytes were considered to be adherent to the venular wall (100 μm of length) if they remained stationary for at least 30 seconds.

### Histological analysis

Brain tissues were fixed in 10% phosphate-buffered formalin, processed and embedded in paraffin. Sections were cut 5 μm and stained with hematoxylin & eosin, and examined under a light microscope by a blinded pathologist.

### Neurobehavioral parameters

CM was defined by clinical evaluation of neurobehavioral parameters based on a multifactorial SHIRPA protocol as described previously [48–50]. CM was identified by the observation of eighteen clinical signs, and to each alteration was given grade one. Above foursigns, including piloerection, curved trunk, alterations in gait, seizures, limb paralysis, coma, respiratory rate, skin color alterations, heart rate, lacrimation, palpebral closure, decreased grip strength, limb, abdominal and body tone and body temperature alterations it was considered clinical CM, that was confirmed by histological evaluation [27].

### Step-down inhibitory avoidance test

The step-down inhibitory avoidance test was performed as we previously described [27]. Animals that were positive for evidence for CM by SHIRPA testing were treated with oral chloroquine (25 mg/kg b.w.) [48], started six days after infection and continued for seven days. Fifteen days post-infection, animals were subjected to behavioral tests. In the training phase, animals were placed on a platform and their latency to step down on the grid with all four paws was measured with an automatic device. Immediately upon stepping down on the grid the animals received a 0.6 mA, 3.0-second foot shock. A retention test trial was performed 1.5 and 24 h after training and the time the animals spent on the platform was recorded.

### Statistics

Statistical analysis was carried out using the GraphPad Prism software (San Diego, CA, USA). P values were calculated using an unpaired Ordinary one-way ANOVA test when result from more than two groups were analyzed among each other, the post test used was the bonferroni’s multiple comparison test. P values were generated using an unpaired T-test when result from only two groups were analyzed. Results are expressed as means ± SEM (median (IQR)). The level of significance was set at P≤ 0.05.

## Results

### *Plasmodium berghei* ANKA infection increases the expression of α_D_ mRNA transcripts in brain tissue

**Fig 1:**
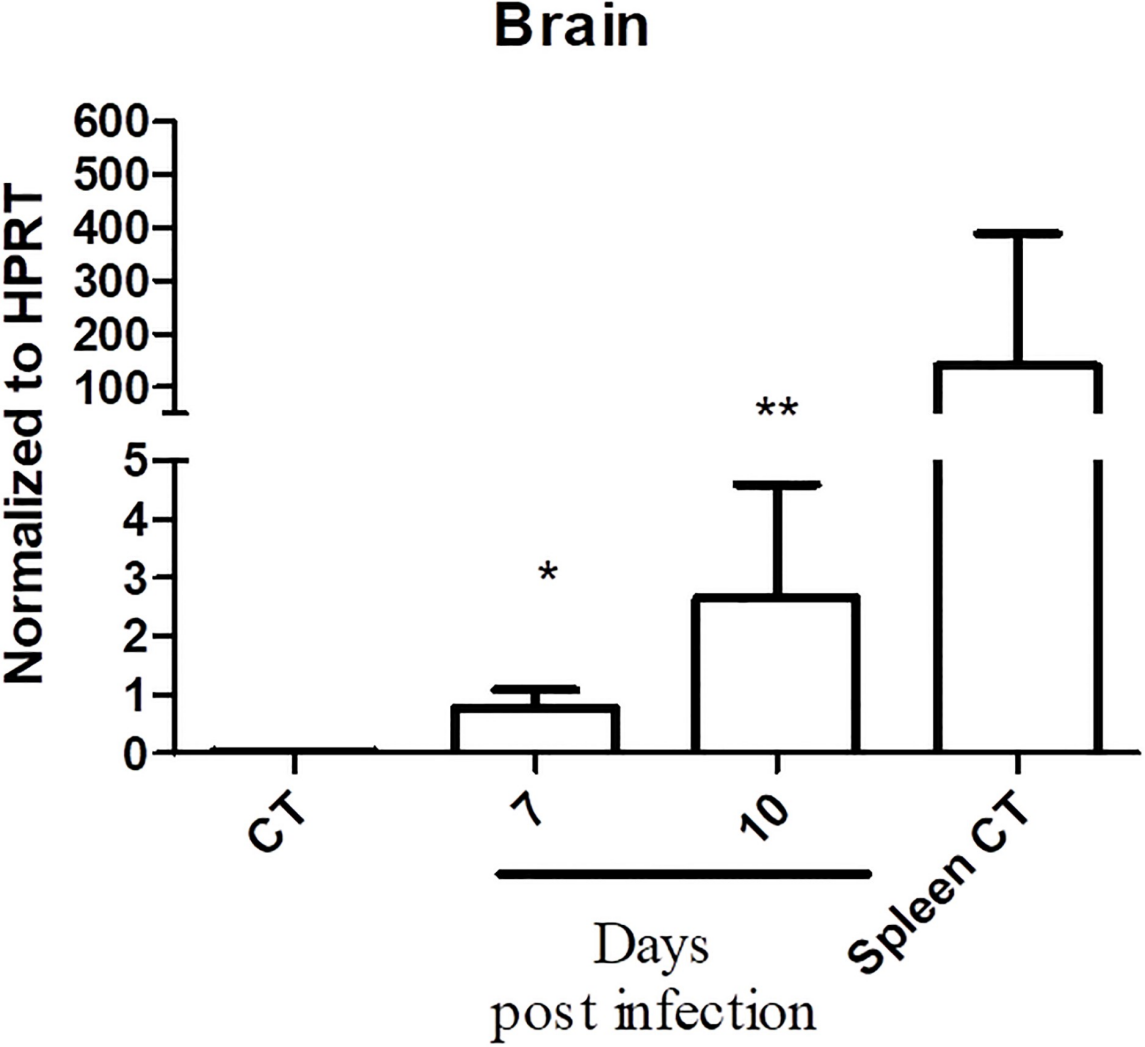
Expression of α_D_ mRNA is increased in the brains of mice infected with *Plasmodium berghei* ANKA. Wild type mice were sacrificed 7 or 10 days post infection. Brains were removed, processed and transcripts for α_D_ were detected and quantified by real-time quantitative PCR and normalized to the levels of hypoxanthine guanine phosphoribosyl transferase (HRPT) as outlined in Methods. Expression of α_D_ in spleen was used as a positive control (Spleen CT; [37]). Each bar represents the mean ± SEM of determinations in tissue from 5–10 animals. *P ≤ 0.04, **P ≤ 0.001 as compared to control (CT) by student’s t test.

*P*. *berghei* ANKA infection in mice is a standard model of experimental cerebral malaria and reproduces most aspects of the human pathology [20, 51, 52]. CM development can be observed to day 6 to 12 post-infection. Accodantly [53, 54], mice that have survived so on presented severe anemia. Once our main interest was the role of α_D_β_2_ integrin, we performed our analysis at day 7 and 10 post-infection. To investigate if *P*. *berghei* ANKA infection modulates α_D_ subunit expression in the brains of infected mice, we examined α_D_ mRNA by real time PCR in brain samples from infected and control animals compared to levels detected in the spleen, where there is high constitutive of integrin α_D_β_2_ on macrophage subpopulations [37]. Real time PCR analysis of uninfected animals demonstrated a low level of the *α*_*D*_ transcript in the brain, consistent with absence of cerebral α_D_β_2_ by immunohistochemical analysis under basal conditions [37]. Expression of the *α*_*D*_ transcript in the brain increased dramatically at 7 and 10 days after infection with *P*. *berghei* ANKA (Fig 1).

### Integrin α_D_β_2_ deficiency alters the abundance of inflammatory mediators in the brain during experimental CM

**Fig 2:**
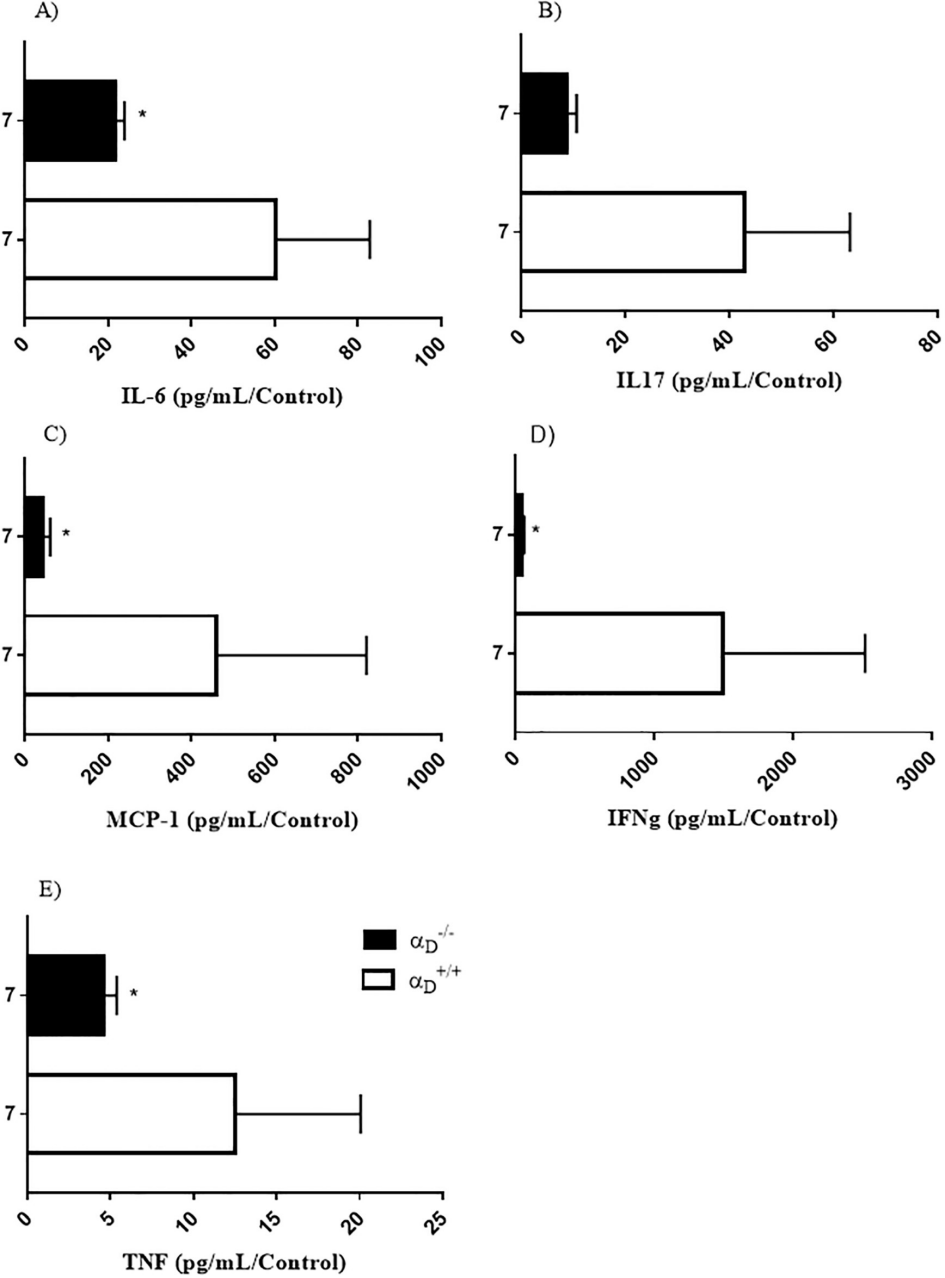
Brain cytokine response to *P*. *berghei* ANKA infection is altered in α_D_^-/-^ mice. Wild type and α_D_β_2_-deficient mice were infected with *P*. *berghei* ANKA and brains were harvested at 7 days post infection. IL6, IFN-g, IL-17, KC, TNF-α and MCP-1 concentrations were quantified by a multiplex assay using a LUMINEX system. Each bar indicates the ratio between the means of infected animals in relation to their corresponding uninfected controls. Were used of 5–10 animals in each experimental group; *P ≤ 0.05 compared to infected wild type (α_D_^+/+^) mice by student’s t test.

The production of inflammatory mediators in the brain is thought to be a critical determinant of CM pathophysiology and neurologic outcomes in severe malaria [21, 22, 55, 56]. We previously found that α_D_β_2_ deficiency alters the patterns of systemic and lung cytokines and chemokines during *P*. *berghei* ANKA infection, leading to a delay in mortality, but without triggering alteration in parasitemia when compared to infected wild animals (S1A and S1B Fig) [37, 38]. Therefore, we measured cytokine and chemokine levels in brain tissue using specific Luminex assays and found increased accumulation of IL-6 (Fig 2A), KC (Fig 2B), IL-17 (Fig 2C), MCP-1 (Fig 2D), IFN-g (Fig 2E) and TNF-α (Fig 2F) in infected α_D_^+/+^ mice as compared to uninfected controls. In contrast, brain cytokine levels in infected α_D_^-/-^ mice were modestly increased when compared to uninfected controls, and cytokines values were significantly decreased in comparison to levels in infected α_D_^+/+^ mice (Fig 2A–2F). These results indicate that regulation of cytokine production and accumulation in the brain is directly or indirectly influenced by α_D_β_2_ in *P*. *berghei* ANKA infection.

### Cerebral edema and leukocyte plugging of microvessels are significantly decreased in α_D_β_2_-deficient mice

**Fig 3:**
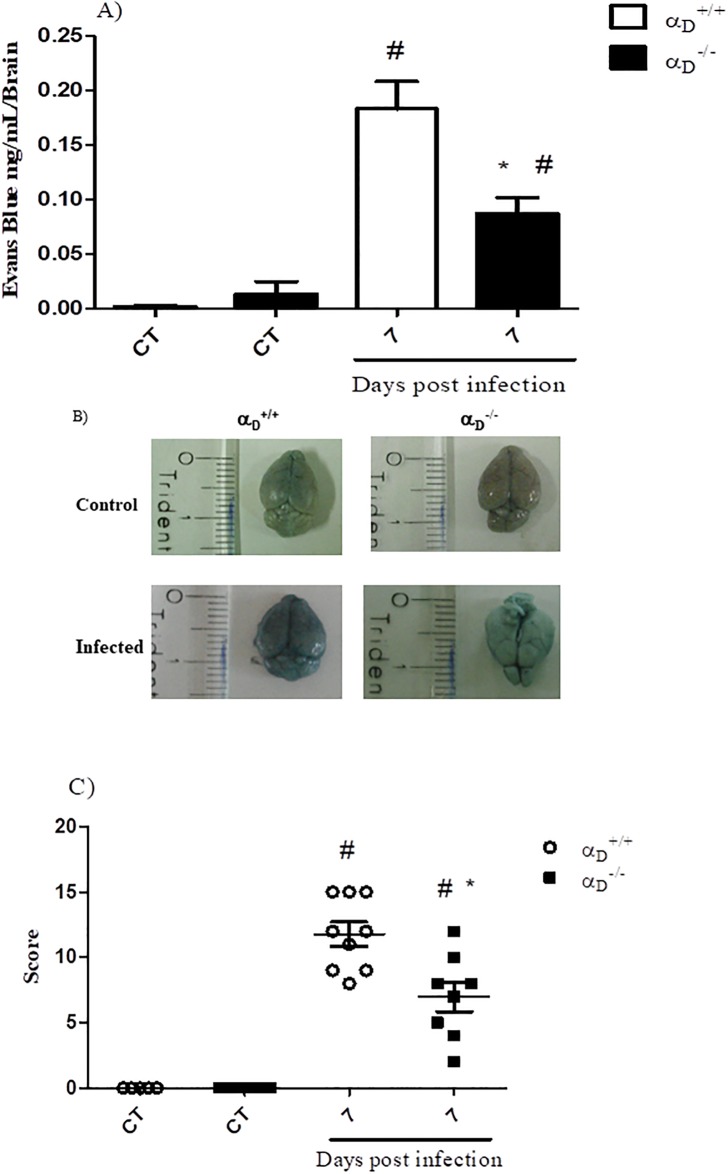
Cerebral edema and clinical signs of CM are decreased in α_D_^-/-^ mice infected with *P*. *berghei* ANKA. Permeability of the blood-brain barrier was determined after injection of 2% (w/v) Evans blue dye solution into the ocular vein. Relative accumulation of Evans blue dye in extravascular brain tissue of α_D_^+/+^ and α_D_^-/-^ animals and scoring of clinical signs of neurologic involvement were analyzed on day 7 post-infection. A) Quantification of dye extravasation. Each bar indicates the mean ± SEM from 5–10 animals. B) images of brains from uninfected α_D_^+/+^ (top left) and α_D_^-/-^ mice (top right); infected α_D_^+/+^ (bottom left) and α_D_^-/-^ (bottom right). C) Clinical score determined by application of the SHIRPA protocol in control (CT) in infected α_D_^+/+^ and α_D_^-/-^ as described in Materials and Methods. Significance was evaluated by ANOVA followed by bonferroni’s multiple comparison test and #P ≤ 0.05 compared to the respective control group; *P ≤ 0.05 compared to α_D_^+/+^ infected mice.

The histology of cerebral malaria is characterized by brain edema, partial or complete occlusion of brain microvessels due to accumulation of host blood cells, micro-hemorrhages and, in some cases, necrosis of the surrounding parenchyma [5, 14, 57, 58]. To assess the status of the blood-brain barrier (BBB) and cerebral edema during *P*. *berghei* ANKA infection, α_D_^+/+^ and α_D_^-/-^ mice were infused with Evans blue dye and brain vascular barrier permeability was examined by quantification of dye extravasation in the brain tissue. We observed a reduction of dye extravasation in α_D_^-/-^ mice as compared to α_D_^+/+^ animals (Fig 3A and 3B) on day 7 post-infection, a time point at which clinical scoring also indicated amelioration of neurological signs of experimental CM in α_D_^-/-^ mice (Fig 3C).

**Fig 4:**
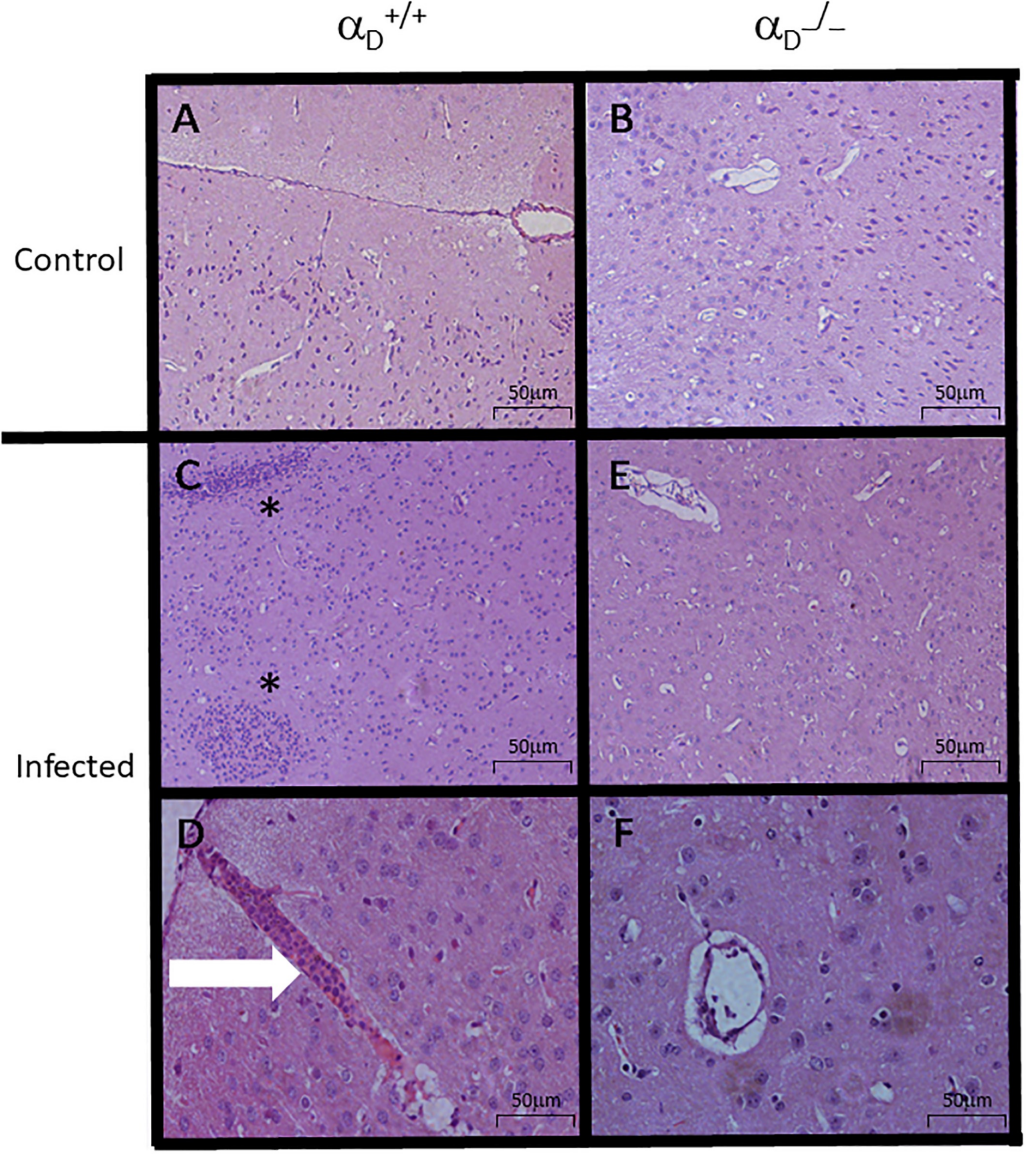
Vascular and parenchymal inflammation are key components of cerebral malaria in *P*. *berghei* ANKA-infected mice and are decreased in α_D_^-/-^ mice. After staining with haematoxylin-eosin, brain sections were examined by light microscopy. The scale bars indicate 50 μm. A) Brain tissue from an uninfected α_D_^+/+^ mouse. B) Brain section from an uninfected α_D_^−/−^ animal. C and D) Brain tissue from an infected α_D_^+/+^ animal. The arrow (Painel D) identifies adherent leukocytes plugging a brain vessel, indicating vascular inflammation. Diffuse interstitial infiltrates (asterisks Painel C) were also seen. E and F) Brain tissue section from an infected α_D_^−/−^ mouse. The features illustrated in A–F are representative of those seen in brain tissue from 3 individual mice of each genotype and condition and 3 independent experiments. Magnifications, x100 (A, B, *C*, and E) and x1000 (*D* and F).

Neurologic signs that herald onset of experimental CM are generally accompanied by the sequestration of leukocytes and infected erythrocytes in the cerebral vasculature [20, 27, 48]. Histological analysis of brain sections obtained seven days post-infection from infected α_D_^+/+^ mice revealed accumulation of leukocytes in brain microvessels, and inflammatory interstitial infiltrates in the parenchyma (Fig 4C and 4D). In contrast, these features were not detected in samples from uninfected α_D_^+/+^ or α_D_^-/-^ mice (Fig 4A and 4B, respectively) or in brain tissue from infected α_D_^-/-^ animals (Fig 4E and 4F). Thus, preservation of BBB integrity in infected α_D_^-/-^ mice (Fig 3) was associated with a decrease in sequestration of inflammatory cells in brain microvessels and with improved clinical scores.

### Integrin α_D_β_2_ deficiency alters leukocyte accumulation in brain microvessels in experimental CM

**Fig 5:**
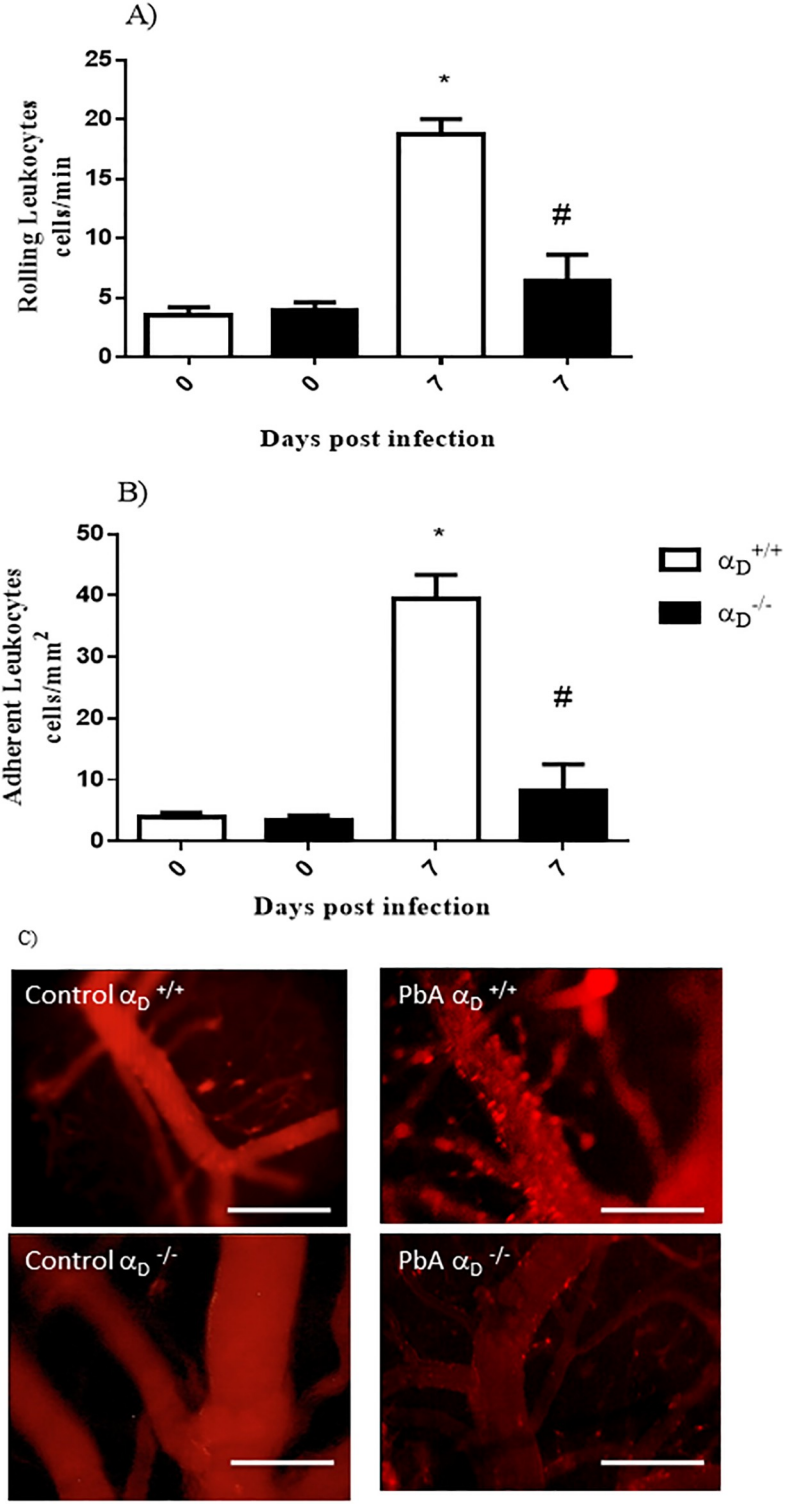
Integrin α_D_β_2_ influences leukocyte-endothelial interactions leukocyte accumulation in cerebral microvessels of mice infected with *P*. *berghei* ANKA. Infected and un-infected α_D_^+/+^ and α_D_^−/−^ mice were analysed at 6 days after infection. Uninfected control (CT) mice were examined in parallel. The numbers of rolling and adherent rhodamine-labeled leukocytes were determined by intravital microscopy during a one-minute period of observation. Panel A indicates analyses of rolling leukocytes and Panel B analyses of adherent leukocytes in three independent experiments. Each bar indicates the mean ± SEM from 5–10 animals. Significance was evaluated by ANOVA followed by bonferroni’s multiple comparison test and * P ≤ 0.05 compared to the respective control group; # P ≤ 0.05 compared to infected α_D_^+/+^ mice. Panel C displays representative images of rhodamine 6G-labeled leukocytes in post-capillary venules in the cerebral microcirculation of *P*. *berghei* ANKA (PbA)-infected α_D_^+/+^ and α_D_^−/−^ mice. Scale bar: 100 μm, 200X magnification. Top panel: Leukocytes adhered to the cerebral venular endothelium of infected wild type (α_D_^+/+^) animals; Bottom panel: Decreased leukocyte adherence in cerebral venules vessels of infected α_D_β_2_ deficient (α_D_^-/-^) animals.

To evaluate contribution of integrin α_D_β_2_ to leukocyte–endothelial interactions in the cerebral microcirculation in *P*. *berghei* ANKA, we examined post-capillary venules displayed through a cranial window by intravital microscopy. At 6 days post-infection, we observed a significant increase (p < 0.05) in rolling (Fig 5A) and adherent leukocytes (Fig 5B and 5C) in α_D_^+/+^ mice when compared to uninfected α_D_^+/+^ or α_D_^-/-^ animals. Rolling and intravascular adhesion of leukocytes in brain vessels of *P*. *berghei* ANKA-infected mice was also detected in another report, and the number of adherent intravascular leukocytes increased as neurologic involvement progressed [59]. Rolling and adhesion were significantly reduced in infected α_D_^-/-^ mice when compared to infected α_D_^+/+^ animals (Fig 5A, 5B and 5C). Venular plugging with leukocytes was detected in α_D_^+/+^ but not α_D_^-/-^ mice (Fig 4D and 4F). Microvascular obstruction may result in dysregulated cerebral blood distribution and hemodynamics in clinical [2] and experimental [22] CM.

### Integrin α_D_β_2_ deficiency reduces CD4^+^ and CD8^+^ T lymphocytes accumulation in the brain during experimental CM

**Fig 6:**
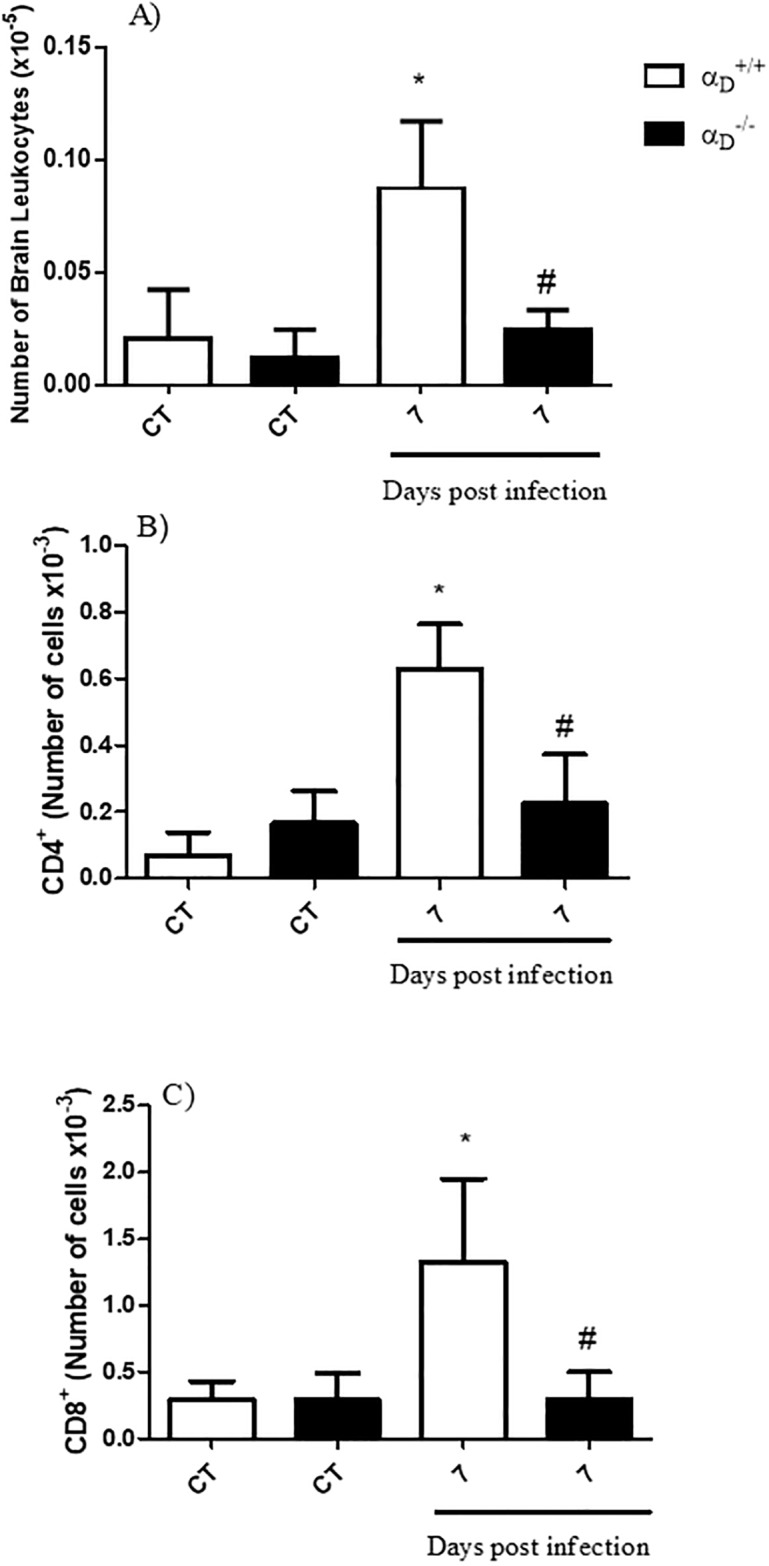
Accumulation of mononuclear leukocyte and CD4^+^ and CD8^+^ T cells is decreased in the brains of infected α_D_^-/-^ mice. At day 7 after infection, brains from α_D_^+/+^ and α_D_^-/-^ mice were harvested, and total mononuclear leukocytes (A) and CD4^+^ (B) and CD8^+^ (C) T cell numbers in cerebral tissue were analyzed by FACS. Brain tissue from control (CT) mice was examined in parallel. Each bar indicates the mean ± SEM of determinations from 5–10 animals. Significance was evaluated by ANOVA followed by bonferroni’s multiple comparison test and *P ≤ 0.05 compared to the respective control group; #P ≤ 0.05 compared to infected α_D_^+/+^ mice in analyses in three independent experiments.

T-lymphocyte accumulation and activity contribute to development of experimental CM [20, 22, 59]. Therefore, we performed flow cytometric analyses of brain cell suspensions from α_D_^+/+^ and α_D_^-/-^ mice at post-infection day 7. As shown in Fig 6A, there was a significant increase in the total number of mononuclear cells accumulating in the brains of α_D_^+/+^ infected mice, consistent with previous reports [22, 59]. The increase in the number of accumulated mononuclear cells was dramatically attenuated in α_D_^-/-^ mice and did not reach statistical significance as compared to uninfected controls (Fig 6A). The number of both CD4^+^ and CD8^+^ T cells was increased in the brains of infected α_D_^+/+^ mice (Fig 6B and 6C). As in analysis of total mononuclear cells (Fig 6A), brain CD4^+^ and CD8^+^ T cell accumulation was substantially reduced in infected α_D_^-/-^ mice. These data indicate that T-lymphocyte accumulation in the brain, a key event in experimental CM pathogenesis [20], may be directly dependent on the expression of integrin α_D_β_2_ during *P*. *berghei* ANKA infection.

### Mice deficient in integrin α_D_β_2_ are protected from CM-induced cognitive impairment

**Fig 7:**
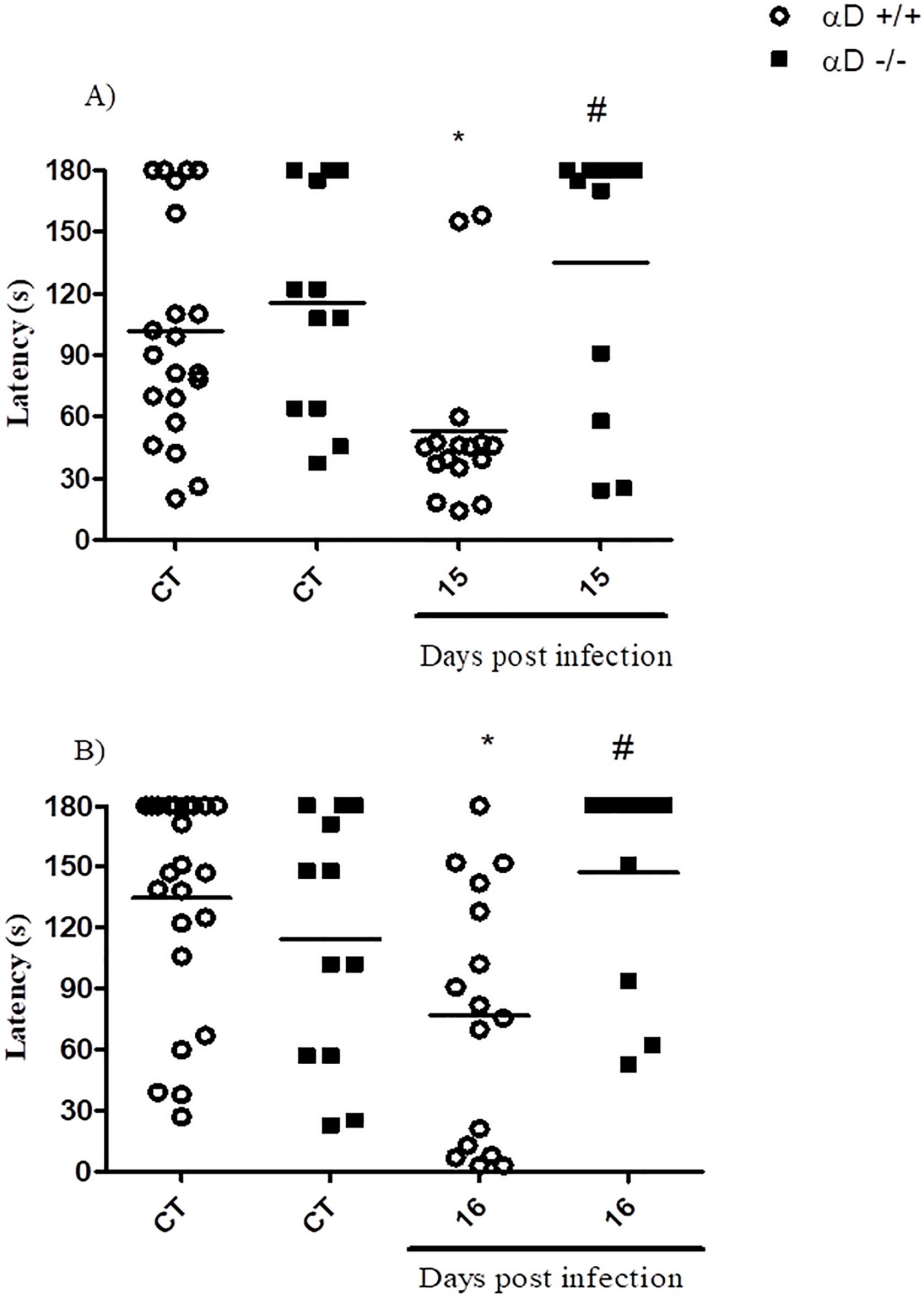
α_D_β_2_ deficiency prevents aversive memory impairment in experimental CM survivors. α_D_^+/+^ and α_D_^-/-^ mice were infected with 10^6^ pRBC. Starting on day 6- post infection, uninfected and *P*. *berghei* ANKA -infected mice were treated orally with chloroquine (25 mg/kg b.w.) for 7 days. On days 15 and 16 post-infection all the animals were examined using a step-down inhibitory avoidance test (Materials and Methods). Uninfected control (CT) mice were examined in parallel. A) 1.5h (Short-term) and B) 24 h (long-term) aversive memory was determined by recording the latency time on the safety platform (with a cut-off of 180 sec). Data are expressed as individual values and horizontal lines represent the mean of latency, in seconds from 12–21 animals. Significance was evaluated by ANOVA followed by bonferroni’s multiple comparison test and *P ≤ 0.05 compared to the respective control group; #P ≤ 0.05 compared to α_D_^+/+^ infected mice.

CM is associated with long-term cognitive impairment in humans [2, 4, 60–63]. Cognitive dysfunction also occurs in animal models [27, 48]. Therefore we asked if the altered leukocyte and cytokine responses we detected in infected α_D_β_2_-deficient mice influence this delayed outcome. We performed experiments in which α_D_^+/+^ and α_D_^-/-^ mice were rescued from experimental CM by treatment with chloroquine initiated after the first clinical signs of CM were detected by the modified SHIRPA score test (Fig 3C), as we have previously described [48]. At days 15 and 16 after infection, surviving animals were examined using an step-down inhibitory avoidance test to assess aversive memory performance. In agreement with our previous observations [27, 48] there was a defect in short and long term aversive memory in infected wild-type mice rescued from CM by chloroquine treatment (Fig 7A and 7B). In contrast, short and long term aversive memory were preserved in integrin α_D_β_2_-deficient animals, and were similar to responses detected in uninfected mice (Fig 7A and 7B).

## Discussion

Severe malaria, a group of complications with organ-specific and systemic manifestations that can result in multiorgan failure, presents differently in children and adults and is commonly caused by *P*. *falciparum* [2, 52, 64, 65]. Among the major clinical complications of severe malaria CM, is most frequent and lethal in children but occurs in all age groups and is a major cause of morbidity and mortality in plasmodial infection [64, 66, 67]. Biologic features of both the parasite and the host influence the severity of malarial infection [1, 66, 67]. An evolving perception is that differential host responses to malaria infection contribute to dysregulated inflammatory effector activities and result in organ dysfunction and injury in CM and other complications of severe malaria [1, 14, 52, 64–67]. Animal models have been important in generating concepts regarding the pathogenesis and natural history of severe malaria and its specific organ involvement [20, 68, 69]. In this study we utilized a murine model that has features of severe human malaria and has been studied extensively as a surrogate for clinical CM—infection of mice of susceptible genotype with the murine malaria parasite *P*. *berghei* ANKA—to provide additional evidence that innate host effector systems contribute to cerebral involvement and to both life-threatening and chronic CNS complications.

We found that targeted deletion of the α_D_ polypeptide integrin subunit, yielding absence of the integrin α_D_β_2_ heterodimer on leukocyte subsets [37], resulted in decreased brain chemokine and cytokine levels, preserved cerebral vascular barrier integrity, blunted intravascular leukocyte accumulation, and decreased numbers of T lymphocytes in brain tissue. Each of these variables is thought to be central in the pathogenesis of severe malaria [14, 20, 65] and is associated with brain inflammation and injury in experimental CM [20, 52, 68, 69]. Consistent with these results, we also found that cognitive function was relatively preserved in integrin α_D_β_2_ “knockout” mice compared to wild type controls when they were rescued by anti-malarial treatment at the onset of signs of neurologic involvement and later studied. These findings in rescued animals indicate that integrin α_D_β_2_ influences key events that alter brain function in experimental CM. Our observations are the first to demonstrate that α_D_β_2_ is an inflammatory effector molecule in CNS infection caused by malaria parasites or other pathogens. It was previously reported that blocking antibodies against α_D_ reduced neutrophil and macrophage numbers and improved cognition and other functional outcomes in experimental traumatic brain injury in rats [70, 71], suggesting that α_D_β_2_ has broad effector activities in both sterile and infectious CNS inflammation.

Decreased brain chemokine and cytokine levels in infected α_D_^-/-^ mice in part mechanistically accounts for preserved blood-brain barrier integrity and reduced vascular and parenchymal inflammation [14, 20, 57]. Disrupted blood brain barrier integrity is a cardinal feature of clinical and experimental CM [2, 22, 64, 67, 72]. Endothelial responses to TNF, which was reduced in the brains of infected α_D_^-/-^ mice (Fig 2), may be particularly important in differentially influencing vascular barrier integrity and other features of severe versus uncomplicated malaria phenotypes [1, 64]. Other proinflammatory cytokines including IFNγ, which was also reduced in brain tissue from *P*. *berghei* ANKA-infected α_D_^-/-^ animals (Fig 2), are implicated as well [20, 65]. In previous investigations we found that integrin α_D_β_2_ regulates *in vivo* systemic [37] and pulmonary [38] chemokine and cytokine levels and that engagement of α_D_β_2_ on human monocytes induces outside-in signals to chemokine and citokynes expression pathways [36]. Thus, interruption of cytokine and chemokine expression in α_D_^-/-^ mice provide a mechanism for amelioration of inflammatory vasculopathy in *P*. *berghei* ANKA-infection animals.

Intravascular leukocyte sequestration reflects cerebrovascular endothelial signaling and circulating leukocyte activation, and is a major feature of experimental malaria [22, 57, 59, 64]. Monocytes, lymphocytes, and platelets are sequestered in brain microvessels and post-capillary venules in mice with cerebral involvement, reflecting key cell-cell interactions [20, 57, 73]. Reduced accumulation of intravascular leukocytes in cerebral microvessels of α_D_^-/-^ mice infected with *P*. *berghei* ANKA- (Fig 5) was one of the most striking findings in our study. Circulating leukocyte numbers are similar in α_D_^-/-^ and wild type animals [37], suggesting that the decreased cerebrovascular leukocyte sequestration in infected knockout mice was due to altered leukocyte adhesion and cellular interactions. Integrin α_D_β_2_ mediates adhesion of murine macrophages and cell lines in static assays [37, 74, 75] and intravascular arrest and retention of macrophages in experimental atherogenesis [39]. Our observations in this study demonstrate for the first time that integrin α_D_β_2_ influences intravascular leukocyte adhesion and accumulation in experimental CM. These events potentially include adhesion of α_D_β_2_-positive leukocytes to inflamed endothelium, activated platelets, iRBC, and/or other leukocytes [22, 31, 33, 59], and remain to be dissected. Our preliminary observations suggest that α_D_β_2_ expression is induced on circulating monocytes [36] which sequester in cerebral microvessels and mediate critical intercellular interactions in experimental CM caused by *P*. *berghei* ANKA infection [59].

Our observation that α_D_β_2_ influences brain CD4^+^ and CD8^+^ T cell recruitment in *P*. *berghei* ANKA infection (Fig 6) is one of the most significant findings from our experiments, given the critical contributions of T lymphocytes, particularly CD8^+^ T cells, to the pathogenesis of experimental CM [20, 59, 68]. CD4^+^ and CD8^+^ T cells sequester in brain blood vessels in *P*. *berghei* ANKA-infected mice [20, 22, 59], and likely were among the rolling and adherent leukocytes that we observed in infected wild type animals. Nevertheless, although integrin α_D_β_2_ is present on subsets of human T lymphocytes [36, 76], it was not detected on lymphocytes from mice of several genetic backgrounds under basal uninflamed conditions [36, 77]. It is possible that α_D_β_2_ is induced on T cells in *P*. *berghei* ANKA infection as the related integrin, α_L_β_2_ (LFA-1), is reported to be [78]. If so, α_D_β_2_ may directly mediate adhesion and intravascular accumulation of CD4^+^ and CD8^+^ T cells in infected animals, events that are abrogated in α_D_β_2_-deficient mice. Alternatively, an indirect mechanism may be involved. Reciprocal interactions between monocytes and T lymphocytes are reported to influence T cell recruitment to the brain in a complex fashion in *P*. *berghei* ANKA infection [59]. Thus, critical signaling events between α_D_β2 –positive monocytes and T cells lacking this integrin heterodimer may occur, and may be interrupted in α_D_β_2_-deficient mice. Futher studies will be required to resolve this issue.

Integrins on leukocytes, and on other relevant cells such as platelets, have intricate activities in inflammation and infection [31, 33, 73]. This report demonstrates that integrin α_D_β_2_ is a functionally important effector in inflammatory events central to experimental CM, including increased blood brain barrier permeability, CD4^+^ and CD8^+^ T lymphocyte accumulation in the brain, and late cognitive impairment in survivors. Blunting of acute brain injury in α_D_β_2_-deficient mice likely contributes to the early survival advantage of α_D_^−/−^ animals in lethal systemic *P*. *berghei* ANKA infection [37], as suggested by improved clinical scores in this study (Fig 3C). Dissecting the roles of leukocyte integrins in CM and other manifestations of severe malaria will provide new insights into the innate and adaptive immune fabric of host responses to Plasmodio, and increased understanding of cell- and organ-specific events in malarial inflammation.

## Supporting information

S1 Fig Deletion of α_D_ provides a survival advantage in *P*. *berghei* infection. Mice were infected by i.p. challenge with 10^5^ *P*. *berghei* PRBC. A) The percentage of RBC infected by *P*. *berghei* was determined by microscopy, results from one of three experiments and B) Two separate experiments, each involving 10 α_D_^+/+^ and 10 α_D_^-/-^ animals, were accomplished and the pooled results were analyzed. No significant differences were observed by Student´s T (A) nor Log-rank (Mantel-Cox) tests (B).
(TIF)

S2 Fig Minimal data set pLOS. Minimum set of data needed to repeat the analyzes procedeed in this paper. We described the number of animals used in each methodology, the mean and error values used to construct the graphs.
(XLSX)
