# Supplementary figures and images for: Integrin αDβ2 influences cerebral edema, leukocyte accumulation and neurologic outcomes in experimental severe malaria
Source: PLoS One. 2019 Dec 23;14(12):e0224610. doi: 10.1371/journal.pone.0224610 (PMC6927624; doi:10.1371/journal.pone.0224610)

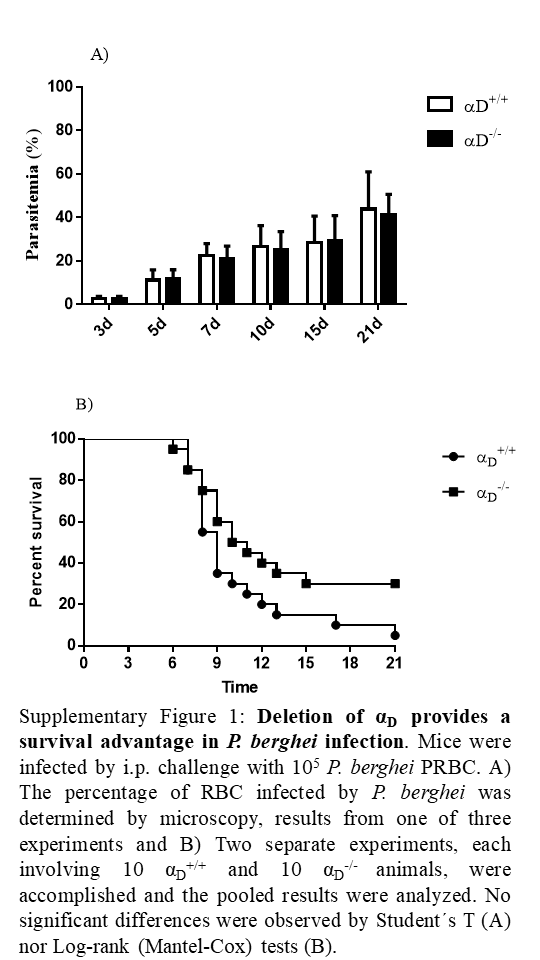

Supplement: S1 Fig — Mice were infected by i.p. challenge with 105 P. berghei PRBC. A) The percentage of RBC infected by P. berghei was determined by microscopy, results from one of three experiments and B) Two separate experiments, each involving 10 αD+/+ and 10 αD-/- animals, were accomplished and the pooled results were analyzed. No significant differences were observed by Student´s T (A) nor Log-rank (Mantel-Cox) tests (B). (TIF) [file pone.0224610.s001.tif]
